# Household air pollution and pneumococcal density related to nasopharyngeal inflammation in mothers and children in Ethiopia: A cross-sectional study

**DOI:** 10.1371/journal.pone.0297085

**Published:** 2024-01-25

**Authors:** Henrik Olsson, Mulugeta Tamire, Ebba Samuelsson, Adamu Addissie, Rune Andersson, Susann Skovbjerg, Simon Athlin

**Affiliations:** 1 Department of Infectious Diseases, Örebro University Hospital, Örebro, Sweden; 2 Department of Preventive Medicine, School of Public Health, Addis Ababa University, Addis Ababa, Ethiopia; 3 Department of Infectious Diseases, Institute of Biomedicine, Sahlgrenska Academy, University of Gothenburg, Gothenburg, Sweden; 4 Department of Clinical Microbiology, Sahlgrenska University Hospital, Region Västra Götaland, Gothenburg, Sweden; 5 School of Medical Sciences, Faculty of Medicine and Health, Örebro University, Örebro, Sweden; Hawassa University College of Medicine and Health Sciences, ETHIOPIA

## Abstract

**Background:**

Three billion people in low- and middle-income countries are exposed to household air pollution as they use biomass fuel for cooking. We investigated the associations between solid fuel use and nasopharyngeal (NP) inflammation, as well as the associations between high pneumococcal density and NP inflammation, in mothers and children in rural and urban Ethiopia.

**Materials and methods:**

Sixty pairs of mothers (median age, 30 years; range, 19–45 years) with a child (median age, 9 months; range, 1–24 months) were included from rural Butajira (n = 30) and urban Addis Ababa (n = 30) in Ethiopia. The cohort was randomly selected from a previous study of 545 mother/child pairs included 2016. Questionnaire-based data were collected which included fuel type used (solid: wood, charcoal, dung or crop waste; cleaner: electricity, liquefied petroleum gas). Nasopharyngeal (NP) samples were collected from all mothers and children and analyzed for the levels of 18 cytokines using a Luminex immunoassay. Pneumococcal DNA densities were measured by a real-time multiplex PCR and a high pneumococcal density was defined as a cyclic threshold (Ct) value ≤ 30.

**Results:**

Mothers from rural areas had higher median CXCL8 levels in NP secretions than those from urban areas (8000 versus 1900 pg/mL; p < 0.01), while rural children had slightly higher IL-10 levels than those from the urban area (26 vs 13 pg/mL; p = 0.04). No associations between fuel type and cytokine levels were found. However, a high pneumococcal density was associated with higher levels of cytokines in both mothers (CCL4, CXCL8, IL-1β, IL-6 and VEGF-A) and children (CCL4, CXCL8, IL-1β, IL-6 and IL-18).

**Conclusions:**

No significant associations were found between solid fuel use and NP inflammation in Ethiopian mothers and children, but the inflammatory activity was higher in individuals living in the rural compared to the urban area. In addition, high cytokine levels were associated with high pneumococcal density in both mothers and children, indicating a significant impact of NP pathogens on inflammatory mediator levels in upper airways.

## Introduction

Three billion people in low- and middle-income countries (LMIC) are exposed to household air pollution (HAP) as they use biomass fuel for cooking and heating their homes. The combustion of solid biomass fuel, i.e. wood, charcoal, crop residues and animal dung, produces high levels of particulate matter (PM) as well as carbon monoxide, nitrogen dioxide and other gaseous molecules [1]. Continuous exposure to airborne pollutants leads to airway cellular damage, increasing the risk of developing airway disease, however, the immunomodulatory effects of HAP exposure in humans are poorly studied [2].

In a recent study, we demonstrated that women in a rural area of Ethiopia using solid fuel for cooking had a higher rate of reduced lung function compared with women using cleaner fuels, i.e. electricity or liquefied petroleum gas [3]. In addition, upper respiratory tract symptoms, including rhinorrhea, sneezing and eye inflammation were more common among the mothers using solid fuel than those using cleaner fuels. In the lower respiratory tract, previous studies have shown induction of inflammatory mediators in lung epithelial cells and alveolar macrophages after exposure to PM [4]. Accordingly, increased cytokine levels in sputum have been demonstrated in women exposed to biomass smoke as a measure of inflammatory activity in the lower airways [5]. However, little is known about the effects of HAP on upper airways in terms of nasopharyngeal (NP) inflammation.

Exposure to HAP may as well lead to pneumococcal colonization in NP due to adhesion of *Streptococcus pneumoniae* to human airway epithelial cells of the mucosa [6]. We have previously shown that NP prevalence of *S*. *pneumoniae* is higher in children and mothers living in rural households using sold fuels than in those living in urban areas using cleaner fuel [7]. Accordingly, individuals exposed to HAP are at increased risk of pneumonia since pneumococcal carriage precedes microaspiration of bacteria from NP to the lungs [8]. However, a need for additional research on the link between HAP exposure and pneumonia, such as modulation of the immune response in the upper respiratory tract, has been suggested [9].

Given that solid fuel is the main energy source for > 90% of Ethiopian households [10], negative effects related to HAP exposure is likely to be a health issue in most areas of Ethiopia, although solid fuel use seems to be more common among rural households [3]. In this study, we investigated the association between solid fuel use, as well as pneumococcal density, and NP inflammation in mothers and children in rural and urban Ethiopia.

## Materials and methods

### Study areas

The study was conducted in the rural area of Butajira, in the East Gurage zone (former Gurage zone), Central Ethiopia Region (former Southern Nations and Nationalities and Peoples Region) in Ethiopia, approximately 136 km south of Addis Ababa, as well as in the urban area of Addis Ababa (Addis Ketema, Gulele and Kirkos sub-cities). The sub-cities were chosen to represent both central and peripheral sub-cities of Addis Ababa. The study areas have been described in detail previously [7]. A majority of households in the rural Butajira area burn biomass materials on open fires as their primary source of energy for cooking, mostly using traditional stoves, while some use locally made improved cook stoves [11]. In urban Addis Ababa, households equally use biomass fuel or cleaner fuel (liquified petroleum gas and electricity) for cooking [3]. Thus, in both areas, mothers spend much of the day close to a lit fire, whereas tobacco smoking is uncommon among adult mothers in the areas and across the country [12].

### Population and data collection

Between March to August 2016, 545 pairs of mothers aged 19–49 years with a child below two years of age were included in a cross-sectional study as described before [3, 7]. Originally, study participants were recruited using systematic sampling technique at health centers located in the rural kebeles of Butajira (mothers/child pairs, n = 279) and the urban kebele of Addis Ababa (n = 266). For this present study, we randomly selected 60 mother/child pairs (rural, n = 30; urban, n = 30) from the original cohort for analyses of cytokine levels and pneumococcal density in NP. Mothers smoking tobacco and those non-resident in the study areas were excluded.

### Baseline characteristics

Baseline characteristics were collected by performing face-to-face interviews with mothers using a questionnaire adopted from the Medical Research Council questionnaire on respiratory symptoms, UK [13], as described before [7]. In brief, the questionnaire was translated to Amharic (national language) with backward translation to English to check for consistency. It contained questions regarding socio-demographic characteristics, primary type of fuels used for cooking, respiratory symptoms in the last 12 months and vaccination status of the children. Solid fuel was defined as wood, charcoal, dung or crop waste, while cleaner fuel was defined as electricity, or liquefied petroleum gas.

### Sample collection from nasopharynx

NP samples were collected from mothers and children at both study sites by trained nurses. In brief, each individual was sampled by inserting a flocked NP swab into the nostril and rotating it over the surface as previously described [7]. The swab was stored in 1 mL of Liquid Amies medium (ESwab^™^, Copan Diagnostics Inc., Murrieta, CA) and transported in ice-boxes to the Bacteriological Laboratory at Tikur Anbessa Specialized Hospital, Addis Ababa University, Addis Ababa. At the laboratory, the samples were frozen at -80°C and stored until transported on dry ice to the Department of Infectious Diseases, University of Gothenburg, Gothenburg, Sweden for further analyses.

### Measurement of inflammatory mediators

Eighteen cytokines were analyzed by performing an 18-plex bead immunoassay (Luminex^®^, Bio-Techne, MN, USA) on a Luminex^®^ 200™ (Bio-Techne) instrument. The following cytokines were measured: C-C motif chemokine ligand (CCL) 2, CCL3, CCL4, C-X-C motif chemokine ligand (CXCL) 8, CXCL10, granulocyte-macrophage colony-stimulating factor (GM-CSF), interferon (IFN)-γ, Interleukin (IL)-1β, IL-6, IL-10, IL-17A, IL-18, IL-33, lipocalin-2 (Lcn2), macrophage colony-stimulating factor (M-CSF), receptor for advanced glycation end products (RAGE), tumor necrosis factor (TNF), and vascular endothelial growth factor (VEGF-A). The assay was performed on undiluted NP samples in duplicates according to the manufacturer’s protocol. The cytokine concentrations were calculated using a log regression standard curve based on expected concentrations of six reference standards for each cytokine ranged (pg/mL): CXCL8: 5–1100; CXCL10: 2–510; GM-CSF: 12–2970; IFN-γ: 51–12490; IL-1β: 20–2970; IL-6: 5–1220; IL-10: 5–1130; IL-17A: 13–3110; IL-18: 10–2460: IL-33: 15–3600; Lcn2: 116–28130: M-CSF: 130–31570; RAGE: 124–30140; TNF: 10–2470; VEGF-A: 9–2090. Concentrations below the lower limit of quantification were set to half the lower limit of detection [14]. Concentrations above the upper limit of quantification were calculated using the standard curve and values out of range were set to the highest calculated concentration.

### Measurement of pneumococcal density

Pneumococcal DNA density was analyzed by performing a real-time multiplex polymerase chain reaction (PCR), which included detection of the *lytA* gene of *S*. *pneumoniae* as previously described [7]. Briefly, after the NP swab medium had been thawed at room temperature, total DNA was extracted from 200 μL medium by a MagNA Pure LC instrument (Roche Diagnostics, Mannheim, Germany) using the Total Nucleic Acid Isolation kit (Roche Diagnostics). The nucleic acids were eluted in 100 μL elution buffer and stored at -20°C until further analyses. A high pneumococcal density was defined as a cycle threshold (Ct) value ≤ 30 while a low pneumococcal density and negative results were defined as a Ct-value > 30.

### Statistical analyses

Cytokine levels and pneumococcal DNA density were compared between individuals living in rural (Butajira) or urban (Addis Ababa) areas of Ethiopia, as well as stratified according to use of solid or cleaner fuel. Seven cytokines with more than 50% of concentrations below the lower limit of detection, namely CCL2, GM-CSF, IL-17A, INF-γ, M-CSF, RAGE and TNF, were excluded from statistical analyses. Descriptive statistics such as mean, median, standard deviation and range for continuous data and frequency for categorical values were calculated separately for each group. Chi-square test, or Fisher’s exact test for cells with an expected count less than five, was used for comparison of pneumococcal DNA Ct-values ≤ 30 and > 30 between groups. Mann-Whitney U-test was used for comparison of inflammatory cytokine levels and results presented as median and interquartile range. A two-tailed p-value of < 0.05 was considered as statistically significant. Statistical analyses were performed using IBM SPSS^®^ Statistics version 27.

### Ethical approval

Ethical approvals were obtained from the Institutional Review Board of the College of Health Sciences of Addis Ababa University, the National Research Ethics Review Committee (NRERC, 3.10/168/2016), Ministry of Science and Technology, Ethiopia and the Regional Ethics Committee in Gothenburg, Sweden (Dnr 115–17). Informed consent was obtained from mothers after written and verbal information had been provided in amharic as approved by the Ethical Committees. Study participants were asked to leave their written consent but the data collector signed the consent document after consent had been obtained verbally in cases of illiteracy. Participation in the study was voluntary and the privacy of participants and confidentiality of the information was assured both during and after data collection. All participants were informed about their right to resign from being part of the study.

## Results

### Baseline characteristics of mothers and children

In the rural area, mothers were older, less educated and used solid fuel as well as suffered from eye irritation to a greater extent, and children were older and had more siblings, than in the urban area (Table 1). The pneumococcal vaccination coverage among children was similar between the two study areas. When mother/child pairs were stratified according to primary fuel type used for cooking, solid fuel was used in 44 (73%) and cleaner fuel in 16 (27%) households, respectively. Solid fuel use was significantly associated with higher age and lower education among mothers compared with cleaner fuel use.

**Table 1:** Socio-demographic characteristics of 60 pairs of mothers and children in rural and urban areas of Ethiopia using solid and cleaner fuel for cooking.

Characteristics	All (n = 60)	Rural (n = 30)	Urban (n = 30)	P-value	Solid fuel (n = 44)	Cleaner fuel (n = 16)	P-value
**Mothers**							
Age, median years (range)	30 (19–45)	35 (24–45)	28 (19–35)	<0.01	31 (19–45)	28 (22–33)	0.04
Education							
No education, n (%)	13 (22)	11 (37)	2 (7)	0.01	13 (30)	0 (0)	0.01
Elementary school, n (%)	30 (50)	18 (60)	12 (40)	0.12	23 (52)	7 (44)	0.56
High school, n (%)	13 (22)	1 (3)	12 (40)	<0.01	7 (16)	6 (38)	0.07
College and above, n (%)	4 (7)	0 (0)	4 (13)	0.11	1 (2)	3 (19)	0.05
Solid fuel, n (%)	44 (73)	30 (100)	14 (47)	<0.01	-	-	-
Respiratory symptoms, n (%)^a^	19 (31)	11 (37)	8 (27)	0.41	15 (34)	4 (25)	0.75
Eye irritation, n (%)	16 (27)	12 (40)	4 (13)	0.04	15 (34)	1 (6)	0.05
High pneumococcal density, n (%)^b^	10 (17)	8 (27)	2 (7)	0.08	10 (23)	0 (0)	0.05
**Children**							
Age, median months (range)	9 (1–24)	12 (2–24)	4 (1–21)	0.02	10 (1–24)	5.5 (1–19)	0.31
Female, n (%)	29	17 (57)	12 (40)	0.20	21 (48)	8 (50)	1.0
Siblings							
No siblings, n (%)	21 (35)	7 (23)	14 (47)	0.06	15 (34)	6 (38)	0.06
One to three, n (%)	29 (48)	14 (47)	15 (30)	0.8	19 (43)	10 (63)	0.19
Four or more, n (%)	10 (17)	9 (30)	1 (3)	0.01	10 (23)	0 (0)	0.05
Solid fuel, n (%)	44 (73)	30 (100)	14 (67)	<0.01	-	-	-
Vaccination, n (%)^c^	52 (87)	24 (80)	28 (93)	0.25	40 (91)	12 (75)	0.19
High pneumococcal density, n (%)^b^	37 (62)	24 (80)	13 (43)	<0.01	30 (68)	7 (43)	0.09

^a^Pneumococcal vaccination according to age.

^b^Defined as Ct-value ≤30 in nasopharynx.

^c^Any of cough, wheezing, phlegm, shortness of breath or irritation of the nose.

### Cytokine levels stratified by rural or urban area

When 11 cytokine concentration levels were compared between individuals living in rural and urban areas, CXCL8 was higher in mothers living in the rural area and IL-10 was higher in children living in the urban area (Table 2).

**Table 2:** Nasopharyngeal cytokine levels among 60 pairs of mothers and children in rural and urban areas of Ethiopia.

	Mothers			Children		
	Rural (n = 30)	Urban (n = 30)	P-value	Rural (n = 30)	Urban (n = 30)	P-value
**CCL3**	360 (290–610)	340 (290–540)	0.92	420 (350–660)	500 (350–630)	0.69
**CCL4**	77 (77–413)	77 (77–390)	0.83	480 (77–720)	460 (77–780)	0.71
**CXCL8**	8000 (2200–10000)	1900 (1000–6800)	<0.01	9900 (5500–10000)	10000 (2600–10000)	0.70
**CXCL10**	150 (89–380)	140 (45–340)	0.50	220 (110–480)	280 (110–4600)	0.44
**IL-1β**	32 (10–290)	10 (10–160)	0.65	530 (110–1500)	220 (10–1200)	0.27
**IL-6**	3 (3–19)	3 (3–22)	0.99	21 (3–28)	15 (3–70)	0.98
**IL-10**	12 (2–34)	15 (9–26)	0.58	13 (2–29)	26 (11–52)	0.04
**IL-18**	32 (5–53)	35 (22–48)	0.58	39 (26–55)	41 (24–51)	0.79
**IL-33**	550 (200–2000)	670 (210–1500)	0.98	460 (260–740)	360 (190–730)	0.64
**Lcn2**	100000 (90000–120000)	86000 (63000–130000)	0.16	100000 (89000–130000)	82000 (61000–140000)	0.06
**VEGF-A**	120 (84–180)	100 (54–210)	0.75	160 (110–220)	150 (77–240)	0.95

Cytokine concentrations are presented as median (interquartile range) pg/mL

### Cytokine levels stratified by use of solid or cleaner fuel

There were no significant differences in cytokine levels between mothers, or children, using solid fuel compared to cleaner fuel (Table 3).

**Table 3:** Nasopharyngeal cytokine levels among 60 pairs of mothers and children using solid or cleaner fuel for cooking.

	Mothers			Children		
	Solid (n = 44)	Cleaner (n = 16)	P-value	Solid (n = 44)	Cleaner (n = 16)	P-value
**CCL3**	350 (270–570)	380 (270–560)	0.81	460 (8400–650)	470 (320–600)	0.59
**CCL4**	77 (77–410)	77 (77–380)	0.75	480 (77–800)	420 (77–560)	0.29
**CXCL8**	4800 (1400–10000)	1700 (820–5400)	0.07	10000 (6000–10000)	7000 (1700–10000)	0.17
**CXCL10**	150 (77–430)	140 (52–220)	0.55	240 (110–580)	250 (99–4100)	0.96
**IL-1β**	10 (10–290)	10 (10–120)	0.63	530 (90–1600)	160 (21–840)	0.19
**IL-6**	3 (3–22)	3 (3–14)	0.61	20 (3–29)	14 (3–45)	0.65
**IL-10**	12 (2–31)	16 (10–18)	0.61	18 (2–37)	21 (11–48)	0.24
**IL-18**	32 (9–50)	40 (10–52)	0.56	40 (25–53)	41 (25–50)	0.97
**IL-33**	620 (220–2000)	670 (230–1100)	0.94	360 (180–720)	490 (290–1000)	0.19
**Lcn2**	100000 (82000–120000)	94000 (68000–1100000)	1.00	97000 (75000–120000)	92000 (68000–910000)	0.95
**VEGF-A**	120 (68–190)	97 (57–190)	0.46	140 (100–230)	160 (75–230)	0.78

Cytokine concentrations are presented as median (interquartile range) pg/mL

### High pneumococcal density and cytokine levels

**Fig 1:**
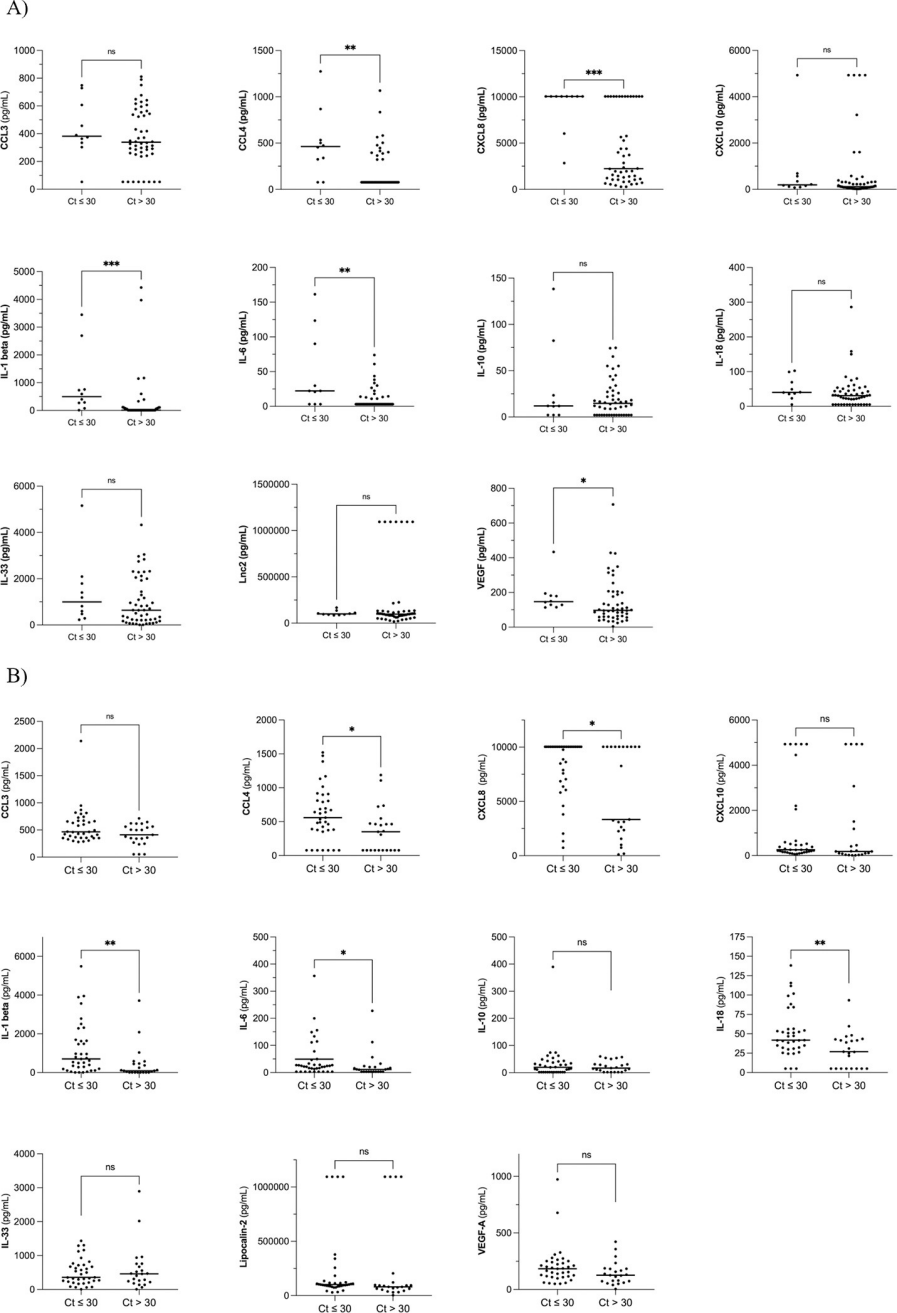
Nasopharyngeal cytokine levels among 60 pairs of mothers (A) and children (B) with high (Ct-value ≤ 30), or low or undetectable (Ct-value > 30) pneumococcal density. Cytokine concentrations are presented as median (interquartile range) pg/mL. Ct, cycle threshold; ns, non-significant.

High pneumococcal densities were detected in 10 of 60 (17%) mothers compared to 37 of 60 (62%; p < 0.01) children. Thirty-four (65%) children adequately vaccinated with PCV13 (n = 52) according to age, and three (38%) unvaccinated children (n = 8), had high pneumococcal densities, respectively. A high pneumococcal density was associated with higher cytokine levels for five cytokines in mothers and for five cytokines in children (Fig 1).

## Discussion

In this study of Ethiopian mothers and children, using solid fuel as primary energy source for cooking was not associated with higher inflammatory cytokine concentrations in upper airways as compared with using cleaner fuel. However, mothers living in the rural area had higher levels of CXCL8 than those living in urban areas. The absence of differences between groups and our findings of cytokine concentrations above those in previous studies on ambient air pollution exposure [15, 16] indicate that other factors than solid fuel exposure may induce inflammation in this population. Instead, the association between high pneumococcal density in nasopharynx and high cytokine levels suggests that the presence of NP pathogens is a significant driver of NP inflammatory mediator production. To our knowledge, this is the first study that focuses on HAP exposure and inflammatory mediators in upper airways, providing results that are useful for future studies.

Initially, we hypothesized that mothers and children living in rural areas of Ethiopia would have higher concentrations of cytokines in upper airways compared with those living in urban areas, due to solid fuel being more commonly used in rural areas as we have previously described [3]. Instead, cytokine concentrations were independent on living area for most cytokines, although the levels were generally higher compared to previous studies on continuous exposure to ambient air pollution in schoolchildren in Taiwan and in children and their mothers in Germany [15, 16]. In mothers, levels of the proinflammatory CXCL8, also known as IL-8, were higher in those living in rural Butajira compared with those living in urban Addis Ababa, but in both groups, the median concentrations were 100 times higher than was found in a previous study of healthy adult volunteers in the Netherlands who were exposed to ambient air pollution during 5 h [17]. In a longitudinal study of children in Taiwan, levels of ambient air pollution were associated with elevated levels of CXCL8 but also in that study the median concentrations were 10–50 times lower than in our study [15]. Higher cytokine concentrations imply a more active induction of NP inflammation in Ethiopian mothers and children in general which may be due to daily exposure to air pollutants from cooking and to heavy ambient air pollution in combination, but more likely from a high burden of NP pathogens [18–20]. Thus, to better understand the impact of solid fuel use on inflammation, our study needs to be repeated using a control group with unexposed individuals and with data on pathogen colonization rates. Furthermore, our finding of higher CXCL8 concentrations in children than in mothers may have various explanations, including increased susceptibility to air pollution exposure in children [16], although higher bacterial pressure in the airways of children is more likely.

Concentrations of the anti-inflammatory IL-10 were slightly higher in children living in urban Addis Ababa compared to rural Butajira. The significance of this finding is unclear and should be assessed in respect of its anti-inflammatory properties [21]. High nasal levels of IL-10 were previously described in a Swedish study of adult volunteers exposed to ambient air pollution in a road tunnel, which suggests an immunostimulatory effect due to air pollution exposure other than from biomass burning [22]. This is supported by a study in Peru, where increased IL-10 levels in blood were negatively associated with exposure to HAP from biomass stoves [23]. However, compared to these previous studies of ambient air pollution exposure, we found high concentrations of IL-10 in all study participants similarly as our findings of high CXCL8 levels. Thus, the small difference in IL-10 between rural and urban areas may be explained by the use of solid fuel for cooking (100% vs 47%), but may also be due to high exposure to ambient air pollution in urban areas such as from diesel exposure [24].

When households were stratified by fuel type, we expected to find higher cytokine levels in the upper airways among solid fuel users compared with those using cleaner fuel, but there were no differences between groups. It is possible that solid fuel is not an important factor behind NP inflammation, however, factors that probably affect our results are the small study cohort, high rates of solid fuel use in both rural and urban areas, a possible use of solid fuel as secondary fuel source in many households, a high degree of exposure to ambient air pollution, and by exposure to gas and liquefied petroleum smoke which was defined as cleaner fuel in this study. Still, to our knowledge, this is the first study of the association between HAP exposure and cytokine levels in the upper airways and may be compared with studies on lower respiratory tract inflammation. Our results contrast to a previous study, in which higher levels of IL-6, CXCL8 and TNF were found in lower airways of rural Indian women who were chronically exposed to smoke from biomass compared to liquified petroleum gas during cooking [5]. Also, several proinflammatory cytokines in serum samples have been associated with biomass smoke [25]. To estimate the impact of solid fuel use on upper airway inflammation, factors listed above need to be considered in future studies, but also including contributing inflammatory activity due to NP pathogens and exposure to solid fuel use in the neighborhood [26] and at work [27].

When we studied the associations between inflammatory activity and pneumococcal density, five pro-inflammatory mediators were significantly higher in mothers (CCL4, CXCL8, IL-1β, IL-6 and VEGF-A), and five in children (CCL4, CXCL8, IL-1β, IL-6 and IL-18), who had high compared with low bacterial densities, respectively. Similar associations between cytokine levels and degree of pneumococcal exposure were found in a recent experimental study of adult volunteers who were inoculated with *S*. *pneumoniae* in NP [20]. Individuals who rapidly cleared the bacteria had elevated levels of MCP-1 and epidermal growth factor (EGF) in NP, while those who had pneumococcal DNA detected in saliva, had elevated levels of CCL3, CCL4, EGF, IL-1β, CXCL8 and hepatocyte growth factor. In cases with established colonization, determined by positive cultures of *S*. *pneumoniae* in NP, cytokine levels were elevated for 12 cytokines, including IL-1β, IL-6 and VEGF-A, for which we found associations with high pneumococcal density in our study. In children, elevated cytokine levels of CXCL8 and TNF have been associated with positive cultures for *S*. *pneumoniae* in NP during acute *otitis media* [28]. Moreover, studies of Swedish children with acute or secretory *otitis media* have shown higher levels of IL-1β and CXCL8 in middle ear fluids if culturable, living bacteria were present, as compared with children without culturable bacteria [29, 30]. In this study, pneumococci were detected by PCR and Ct ≤30 was defined as high pneumococcal density which most likely corresponds to culturable, living bacteria in a majority of cases [31]. Thus, in accordance with previous findings, our results suggest that inflammatory activity is associated with the degree of bacterial exposure in this population, which needs to be addressed in future studies on the impact of solid fuel use on respiratory immune modulation [9].

There are several limitations of this study. First, a possible impact of the households´ solid fuel use may have been concealed by exposure to other sources of pollution, including ambient air pollution, as already discussed. Also, the households that reported a use of electricity as primary energy source for cooking may be exposed to biomass smoke as they experience power blackouts regularly. In addition, the numbers of mothers and children included in the study were small, which reduced the possibility to demonstrate differences between groups. Therefore, new studies need be conducted on larger cohorts and with non-exposed control groups included. Second, the stratification according to fuel type used was performed retrospectively, since the original inclusion was conducted for equal distribution between rural and urban areas of Ethiopia [3]. Since multiple fuel use is common in urban Addis Ababa [32], we performed a redistribution of households according to fuel type used, although a prospective stratification by fuel type would have been preferred. Third, the presence of pathogens is a major driver of inflammatory activity in NP and probably conceals possible impact of solid fuel use in Ethiopian households. Therefore, we used pneumococcal density to estimate the impact of bacterial derived inflammation, since *S*. *pneumoniae* has highly immunogenic properties on the mucosa, but other airway pathogens may induce inflammation as well [18, 19]. Finally, potential differences between groups were probably concealed by cytokine measurements outside limits of quantifications. Since this is the first study of the immunomodulatory effects of solid fuel use on upper airways, we believe that our results will guide the selection of pre-specified ranges for analytes in future studies.

In conclusion, no significant associations were found between solid fuel use and NP inflammation in Ethiopian mothers and children, but the inflammatory activity was higher in individuals living in the rural compared to the urban area. In addition, high cytokine levels were associated with high pneumococcal density in both mothers and children, indicating a significant impact of NP pathogens on inflammatory mediator levels in upper airways.

## Supporting information

S1 Checklist Strengthening the Reporting of Observational Studies in Epidemiology (STROBE) checklist. (PDF)
